# Preclinical Performance Evaluation of a Robotic Endoscope for Non-Contact Laser Surgery

**DOI:** 10.1007/s10439-020-02577-y

**Published:** 2020-08-12

**Authors:** D. Kundrat, R. Graesslin, A. Schoob, D. T. Friedrich, M. O. Scheithauer, T. K. Hoffmann, T. Ortmaier, L. A. Kahrs, P. J. Schuler

**Affiliations:** 1 Leibniz Universität Hannover, Institute of Mechatronic Systems, Appelstraße 11a, 30167 Hannover, Germany; 2 Hamlyn Centre for Robotic Surgery, Imperial College London, London, SW7 2AZ UK; 3 Department of Otorhinolaryngology, Head and Neck Surgery, Ulm University Medical Center, Frauensteige 12, 89075 Ulm, Germany; 4 Surgical Oncology Ulm, i2SOUL Consortium, Ulm, Germany; 5 Department of Otorhinolaryngology, Head and Neck Surgery, Augsburg University Medical Center, Stenglinstr. 2, 86156 Augsburg, Germany; 6 Department of Mathematical and Computational Sciences, University of Toronto Mississauga, Mississauga, ON L5L 1C6 Canada

**Keywords:** Continuum robot, TORS, Endoscopy, Haptics, Head, Neck, Ablation, Performance, Tracking, Motion compensation

## Abstract

Despite great efforts, transoral robotic laser surgery has not been established clinically. Patient benefits are yet to be proven to accept shortcomings of robotic systems. In particular, laryngeal reachability and transition from microscope to accurate endoscopic laser ablation have not been achieved. We have addressed those challenges with a highly integrated robotic endoscope for non-contact endolaryngeal laser surgery. The current performance status has been assessed in multi-level user studies. In addition, the system was deployed to an *ex vivo* porcine larynx. The robotic design comprises an extensible continuum manipulator with multifunctional tip. The latter features laser optics, stereo vision, and illumination. Vision-based performance assessment is derived from depth estimation and scene tracking. Novices and experts (*n* = 20) conducted teleoperated delineation tasks to mimic laser ablation of delicate anatomy. Delineation with motion-compensated and raw endoscopic visualisation was carried out on planar and non-planar nominal patterns. Root mean square tracing errors of less than 0.75 mm were feasible with task completion times below 45 s. Relevant anatomy in the porcine larynx was exposed successfully. Accuracy and usability of the integrated platform bear potential for dexterous laser manipulation in clinical settings. Cadaver and *in vivo* animal studies may translate *ex vivo* findings.

**Electronic supplementary material:**

The online version of this article (10.1007/s10439-020-02577-y) contains supplementary material, which is available to authorized users.

## Introduction

The high scientific interest in robot- and computer-assisted systems in head and neck surgery has been pushing technologies over the recent years. However, these systems still need to find their way into daily clinical practice.^12^ One main objective in any surgical approach is to perform the procedure with minimal trauma to the concerned region to avoid loss of function. Especially in oncological treatments this course of action may need adjustment to ensure optimal tumor resection. The larynx represents an exceptional position, not only with its challenging anatomy but also due to its impact on life quality when harmed. With increasing indications for robot-assisted surgery (RAS) multiple systems are under development and some have already demonstrated feasibility in preclinical and clinical trials.^28^ Today, the DaVinci system (Intuitive Surgical, Sunnyvale, USA) plays a leading role, even in the head and neck region, and potential indications are successively expanded. Numerous transoal robotic surgeries (TORS) have been undertaken in difficult-to-reach sites such as the hypopharynx and larynx.^4^ Alternatively, the Flex® system (Medrobotics, Raynham, USA) provides an FDA-approved non-rigid robot that can adjust to the anatomy of the pharynx.^29^ In contrast to RAS, laser surgery has been firmly established. It is best known for its medical application in eye surgery but is also used in urology or gynecology.^2^,^7^,^13^ In head and neck surgery, lasers have been introduced in the larynx and are nowadays frequently used for transoral resection of pharyngeal or laryngeal tumors as an alternative to open surgery.^30^ But also in phono-microsurgery of the vocal cords, lasers are beneficial for patients as the procedure takes place in the delicate laryngeal setting.^24^

First attempts to combine both RAS and laser delivery used the DaVinci system and a CO_2_-laser.^6^ In the conventional surgical laser setup, the patient is positioned on its back with extension of the neck. Thus, ensuring a direct view on the glottic region by inserting a rigid laryngoscope into the oral cavity. A microscope magnifies the situs and a manual micromanipulator enables laser steering. In comparison to micro instruments the use of lasers ensures improved organ preservation and accurate incisions.^8^ The conventional setup was upgraded with a motorised laser micromanipulator, a touchscreen, and a graphics stylus.^19^ This automation decreases coordination deficiencies and enhances surgical skills.^10^ However, executable workspaces are limited, visual assessment of the procedure may be impeded due to tissue obstructions, and anatomical access may be restricted due to spinal immobility and dimensional constraints of external scanning units. Those problems have motivated endoscopic laser delivery. For example, related work comprises piezo-electric micro-scanners^22^ embedded to the tip of an endoscope,^16^ magnetic fibre scanners,^1^ or Risley-prisms.^21^Nontheless, workspaces are still limited due to actuation principles, focused radiation is disregarded, field of views are restricted, or manipulation accuracies are insufficient for soft tissue surgery.

**Figure 1:**
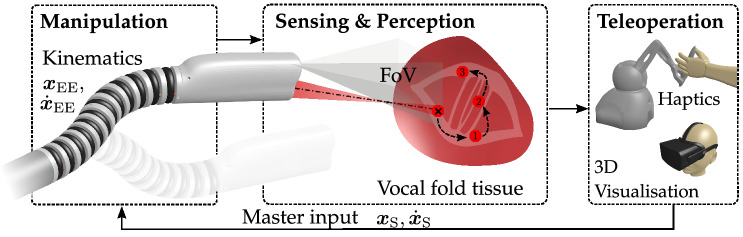
(a) Schematic setup of robotic laser-assisted lesion delineation on vocal fold soft tissue. The beam is displaced from the posterior (1) to the anterior (3) section of the vocal fold while following the vibratory edge (2). The intervention is monitored within the field of view (FoV) of the endoscopic camera. Teleoperation, 3D visualisation, and haptic feedback are taken into consideration for robot control.

We address those problems differently with miniaturised fix-focus laser optics^15^ deflected by a dexterous extensible endoscopic robot.^17^ This approach not only provides increased degrees of freedom (DoF) for beam manipulation compared to related work but also enables to address the problem of laser focus adjustment to the tissue surface in endoscopic settings. This novel combination of RAS and non-contact endoscopic surgery confronts surgeons with unfamiliar procedural workflows. Hence, our prior work has revealed significant advantages of visuo-haptic assistance for teleoperated endoscopic focal adjustment in a user study on an early prototype of our robotic platform.^17^ However, teleoperation was restricted to one DoF only, i.e. adaptation along the beam propagation axis. Beyond workflow integration of spatial laser properties, accurate laser delineation of the targeted lesion is mandatory for clinical routines. More precisely, the focused laser beam must be displaced and positioned with high accuracy (< 1 mm) to preserve delicate anatomy and maintain obtain high energy densities at the tissue surface.^19^ To translate those clinical demands to non-contact endoscopic laser procedures, we propose a dexterous robotic framework with extensible continuum manipulator for luminal deployment as depicted schematically in Fig. 1. The device not only facilitates concurrent endoscopic beam manipulation and focus adaptation for the first time but also may gain access to luminal anatomy through natural or artificial orifices (< 12 mm diameter) and exposure of landmarks with integrated vision. As shown in Fig. 1, kinematic manipulation of the laser beam simultaneously displaces vision sensors. In order to provide clinical users with a familiar microscopic view, e.g. such as in phono-microsurgery, sensor motion is compensated online with tracking and imaging warping methodologies to imitate stationary imaging sensors.

Addressing those challenges and providing comparability with related work, we hypothesize that subjects from different clinical experience levels can achieve target delineation errors of less than 1 mm with teleoperation of the laser beam by proposed endoscopic master–slave robot. Additionally, the problem of joint beam displacement and focus adjustment is targeted in the first reported case study on assisted 3 DoF beam manipulation on non-planar surfaces with our proposed platform. A step towards clinical translation is further provided by a deployment to laryngeal porcine model to assess handling, visualisation, and endoscopic dexterity in an *ex vivo* scenario.

Study metrics are computed from vision sensors, subsequent depth estimation, and scene tracking. In summary, this work presents the first fully integrated robotic endoscope (5 DoF total) that enables endoscopic delivery of focused laser radiation under joint consideration of kinematics-based beam displacement and focus adjustment. The corresponding motion of embedded vision sensors is compensated by a tracking and warping workflow to provide familiar surgical views. The performance and applicability of proposed designs and methodologies for lesion delineation is evaluated in an experimental phantom study with 20 subjects. An advanced case study of 3 DoF path delineation on non-planar surfaces with three trained subjects and deployment to a porcine larynx underline the advantages of the proposed extensible continuum robotic framework in comparison to scanner-based approaches in related work.

Such technology may pave the way for atraumatic endoscopic non-contact laser ablation in confined anatomy.

## Materials and Methods

This section comprises six subsections: (1) robotic framework, (2) user study design, (3) image-based measurements, (4) evaluation methodology, (5) statistics, and (6) *ex vivo* animal model.

### Robotic Framework

**Figure 2:**
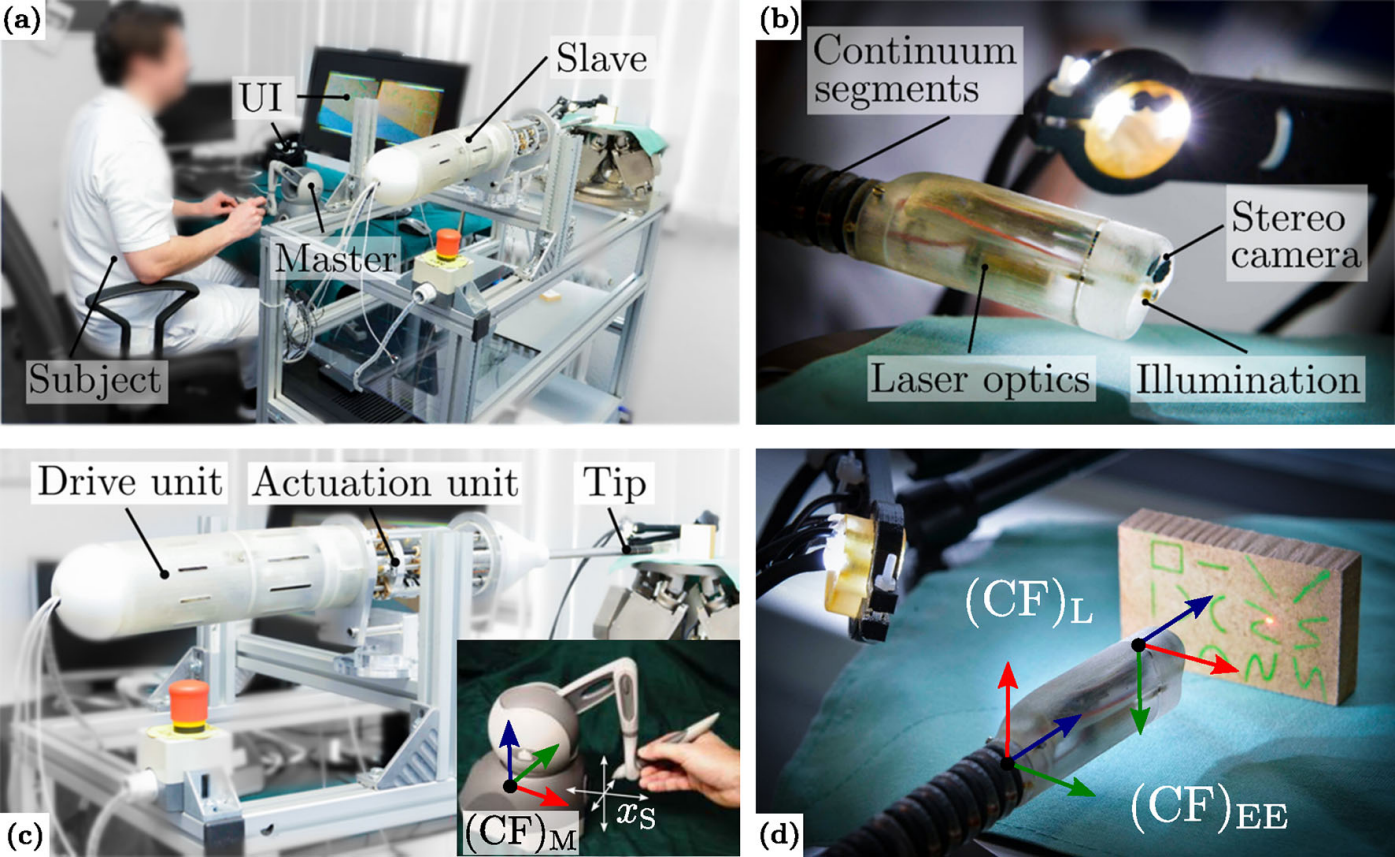
(a) Subject operating the slave robotic with the haptic master device. The user interface (UI) enables stereoscopic image rendering through stereoscopic monitor or head-mounted display. Side-by-side images are for demonstration purposes only. (b) Actuated endoscopic distal tip attached to flexible continuum segments with integrated stereo vision, illumination, and fix-focus laser optics. (c) Slave robot mounted to frame with drive unit, actuation unit, and tip. The overlay image shows the master device with corresponding reference frame of the stylus. (d) Magnified view of the endoscopic tip facing a target with nominal ablation patterns.

A master–slave configuration is considered for robotic teleoperation of the endoscopic tip as shown in Fig. 2a. The slave features an extensible hollow-core continuum manipulator (diameter of 11 mm) for implementation of 5 DoF at the end-effector, a bespoke endoscopic tip (see Fig. 2b) with stereo vision, illumination, and fix-focus laser optics with fibre-based radiation transmission at 2.94 µm wavelength.^15^ Contrary to related work, observed displacement of the laser corresponds to scene motion due to an eye-in-hand camera configuration. The continuum manipulator is manufactured monolithically and is composed of two flexible segments. Each segment is individually actuated by three polyamide tubes which are guided concentrically to the adjacent segment. Coordinated push–pull actuation of six tubes in joint space is implemented with actuation and drive units (see Fig. 2c). The latter mount servo drives commanded by a kinematic controller. Tube motion translates to bending, compression, and extension of the manipulator. Bending angles of approx. 90 degree per segment and extension up to 80% from initial configuration are feasible. The reader is kindly referred to previous work for further details on the robotic hardware.^17^ The kinematic controller extends an established kinematic model 5 to non-constant segmental lengths. Accordingly, direct, inverse, and instantaneous kinematics for bidirectional mapping of end-effector position $\varvec{x}_{\text{EE}} \in {\mathbb{R}}^{5}$ and velocities $\dot{\varvec{x}}_{\text{EE}} \in {\mathbb{R}}^{5}$ to actuation unit joint space ${\varvec{q}},{\dot{\varvec {q}}} \in {\mathbb{R}}^{6}$ are derived. Corresponding coordinate frames (CF) are indicated in Figs. 2c and 2d. Sequence A of the supplemental video demonstrates an example of manipulator dexterity and kinematics. Operator input $\varvec{x}_{\text{S}} \in {\mathbb{R}}^{3}$ is captured by a haptic master (Phantom Omni, 3D Systems, USA) and downscaled by a factor of two. Although the robotic end-effector enables manipulation in 5 DoF (see sequence A), master input in this study is constrained to three linear DoF. The angular input is disregarded due to absence of haptic torque feedback for angular axes of the employed master as well as operational constraints from laryngeal deployment. This prevents generation of additional cognitive loads to the operator due to master manipulation with haptic feedback only on a subset of available DoF. Rendered haptic feedback for linear DoF is generated equivalently to prior work.^17^ This feedback renders a viscous friction model to corresponding master axes that moderates operator input velocities. In this regard, temporal pose offsets between master and slave are mitigated and structural integrity is preserved due to suppression of highly-dynamic inputs. Workspace limits are indicated to the user through vibrational bursts. As shown in Fig. 2a, endoscopic images are visualised on a stereoscopic monitor (VG278H, ASUSTeK Computer Inc., Taipeh, Taiwan) or head-mounted display (Oculus Rift DK2, Oculus VR, Irvine, CA, USA) with tailored user interfaces (UI). Online scene depth recovery with root mean square errors (RMSE) of 0.1 mm is achieved by a reconstruction algorithm.^25^ Estimation of scene motion and deformation is implemented with a tracking algorithm that is optimised for soft tissues with errors of less than 0.1 mm.^26^ The robotic eye-in-hand setup with rigid laser optics integrated to the tip causes endoscopic visualisation with scene motion while displacing the laser spot. This is different from familiar scenarios in transoral laser surgery, where the beam is displaced on a static scene in the microscopic view. For comparative reasons, the latter is mimicked and translated to the robotic scenario. Online fusion of tracking data and endoscopic live images into a warping framework enables motion-compensated scene views^27^ as demonstrated in sequence B of the supplemental video. Vision, haptics, and kinematics are unified in the robot operating system (ROS) framework with update rates of 25 Hz, 1 kHz, and 100 Hz, respectively. Algorithmic processing of vision data is parallelised with GPGPU acceleration (GeForce GTX Titan, NVIDIA Corporation, Santa Clara, CA, USA) on a dedicated workstation.

### User Study Design

The study considers quantitative assessment of the device performance in a preclinical setting that mimics a clinical routine. A custom task was designed to reproduce the workflow of laser-based soft tissue resection. A predefined planar nominal path must be delineated with teleoperated manipulation of a visible laser. Concurrently, two different scene visualisations are rendered to determine effects on metrics based on the eye-in-hand camera layout. Those comprise motion-compensated and raw endoscopic images. The robot was manipulated in three DoF to implement tracing motion and initial adjustment of the distance to the specimen. Participants received written information and gave consent for study participation as well as post-experimental data analysis. Studies were conducted according to ethical standards of Leibniz Universität Hannover. No high-power ablation was performed due to local safety policies. Substitution was provided with a red pilot beam (FLEXPOINT MINI, Laser Components GmbH, Olching, Germany).

The supervisor introduced participants to the experimental procedure. Subjects were asked to select a visualisation modality, i.e. 3D monitor or head-mounted display. An induction of five minutes was granted. This comprised robotic hardware and laser displacement along nominal paths. The latter and visualisation conditions were randomised prior to each series with five repeated measurements per visualisation condition (*n *= 10). Selections from the four path shapes (line width 1 mm) in Fig. 2d must satisfy an equal drawing distribution. In the beginning of each experiment, the supervisor relocated the robot, i.e. the laser spot, to the vicinity of the selected path. Subsequently, a region of interest (RoI) enclosing the selected path was defined on the live image in the UI to initialise image-based measurements. The subject triggered the start of the measurement by activating the pilot laser with a foot pedal. The master device was released with a stylus button and the operator started completion of the tracing task with minimal path error and duration. Task termination was triggered by executing previous steps in reverse order. The robot was teleoperated in three DoF, whereas the initial phase considered assisted adaptation of the tissue distance in only one DoF. If the distance adjustment reached a threshold of less than ± 1 mm, axial displacement of the master was locked, and evaluation measurements were started. Hence, lateral motion in two DoF was feasible to trace the nominal pattern. This sequence was repeated until randomised series were completed.

In contrast, clinical scenarios commonly comprise curved tissue surfaces that must be considered in dexterous manipulation. In this regard, a case study on 3D path delineation has targeted full manipulation in 3 DoF on non-planar specimens with pyramidal relief (see Fig. 9a). Skilled operators were provided with motion-compensated visualisation and absence of constraints on axial displacement. Visuo-haptic assistance^17^ (VHA) has been augmented to fundamental haptics introduced in “Robotic Framework” section to support the focal adjustment. For comparative reasons, trials were repeated with no assistance (NA).

Data acquisition for composition of task metrics comprised: (1) recording of master/slave kinematics and image streams, (2) initial vision-based detection of the coloured path for each task, and (3) continuous vision-based detection of the laser centroid. Robot and image data were acquired at 100 Hz and 25 Hz, respectively. Subjects were asked to complete a user experience survey. The questionnaire in Table 1 combines the task load index protocol (TLX) and after-scenario questionnaire (ASQ).^14^,^18^ A list of 14 statements was developed and rated on a five-point ordinal Likert scale ranging from rejection to agreement.

**Table 1:** Questionnaire for post-experimental task evaluation.

No	Statement	Score
1	The system is easier to operate with image stabilization activated than without	R (0)–A (4)
2	The system is easy to operate	R (0)–A (4)
3	The application of the system is precise	R (0)–A (4)
4	I need more time for familiarization with device and application	R (0)–A (4)
5	I think I need the support of a technician for device operation	R (0)–A (4)
6	I find the system unnecessarily complex	R (0)–A (4)
7	I think I would use the system frequently	R (0)–A (4)
8	I think the system functions are well integrated	R (0)–A (4)
9	I am satisfied with my performance in the given task	R (0)–A (4)
10	The application of the system is safe	R (0)–A (4)
11	I would recommend the system to peers	R (0)–A (4)
12	I felt stressed or bored with this task	R (0)–A (4)
13	The use has made me physically tired	R (0)–A (4)
14	The technical characteristics of the system were sufficient to complete the task	R (0)–A (4)

Rejection (R) and acceptance (A)

### Image-Based Measurements

This section describes visual measurements for assessment of delineation errors. The procedure addresses the initial detection of the nominal path and the continuous detection of the laser spot. The eye-in-hand camera layout motivates the use of integrated stereoscopic sensors in combination with scene reconstruction and tracking.

## Nominal Path Detection

**Figure 3:**
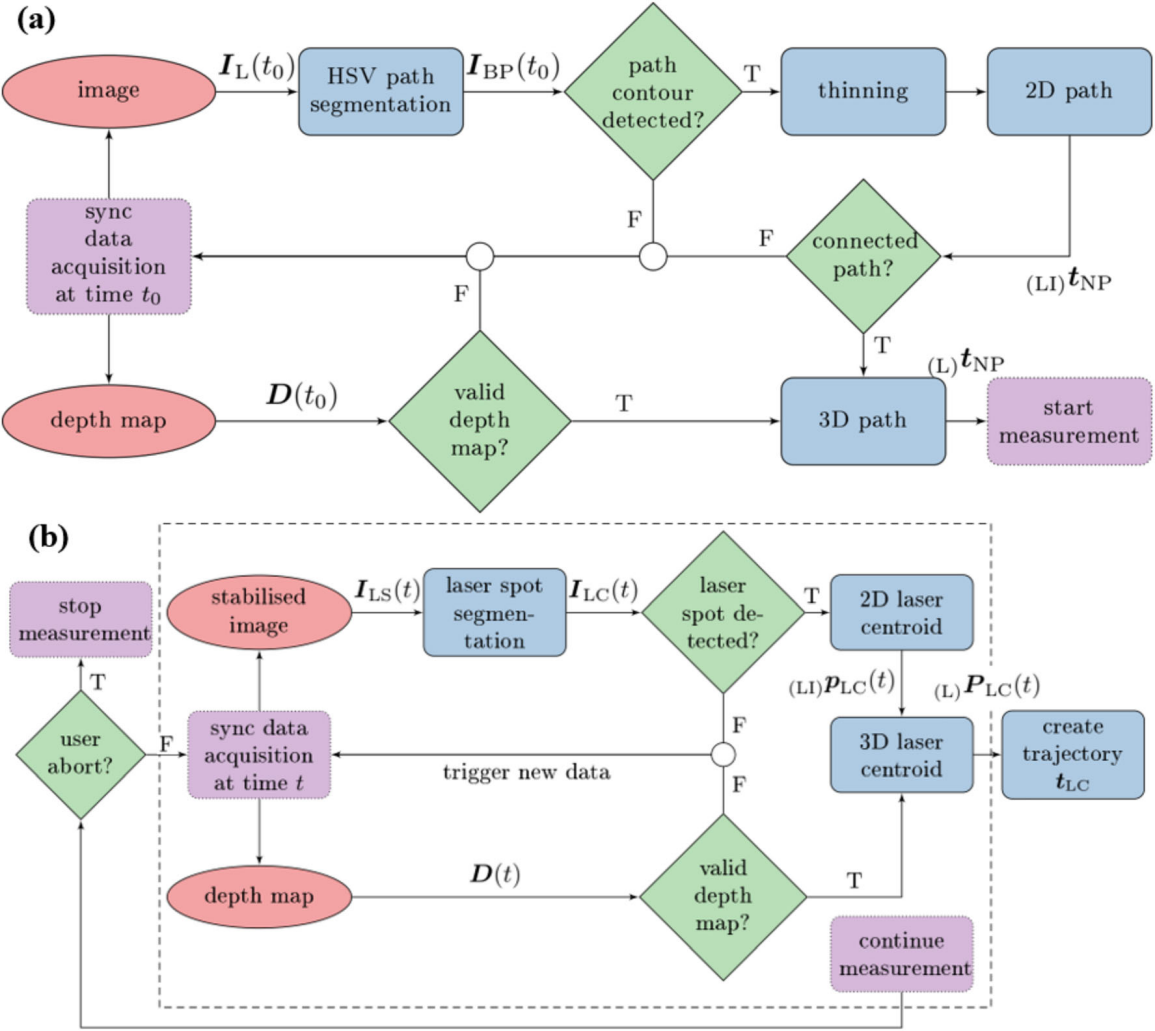
Sequence diagrams of visual measurements: (a) Image processing for nominal path detection, segmentation, extraction, and mapping from image to 3D space. (b) Laser spot segmentation and spatial mapping for consecutive measurements of the spot location during execution of the user task. Acronyms are defined as follows: false (F) and true (T).

The detection workflow builds upon stereoscopic imaging and RoI selection of the nominal coated path in accordance with the experimental protocol in “User Study Design” section. Thereupon, the RoI encloses the nominal path and the processing workflow depicted in Fig. 3a is triggered at time step $t_{0}$ of each experiment.

Primarily, stereoscopic image acquisition and depth map processing are executed. Left image $\varvec{I}_{\text{L}}$ is converted to HSV (Hue-Saturation-Value) space and colour thresholding is applied with respect to visual properties of the path coating. The resulting binary image $\varvec{I}_{\text{BP}}$ is analysed morphologically for connected areas. Afterwards, a thinning algorithm 34 is applied to the dilated area to obtain nominal trajectory ${}_{{\left( {\text{LI}} \right)}} \varvec{t}_{\text{NP}} = \left( {{}_{{\left( {\text{LI}} \right)}} \varvec{p}_{{{\text{NP}},1}} ,{}_{{\left( {\text{LI}} \right)}} \varvec{p}_{{{\text{NP}},i}} , \ldots ,{}_{{\left( {\text{LI}} \right)}} \varvec{p}_{{{\text{NP}},M}} } \right)$ composed of points ${}_{{\left( {\text{LI}} \right)}} \varvec{p}_{{{\text{NP}},i}} = \left( {{}_{{\left( {\text{LI}} \right)}} u_{{{\text{NP}},i}} ,{}_{{\left( {\text{LI}} \right)}} v_{{{\text{NP}},i}}  } \right)^{\text{T}} \in {\mathbb{N}}^{2}$ given in image space (CF)_LI_ with $i = \left\{ { 1, \ldots ,M} \right\}$, where *M* defines the total number of segmented points. Subsequently, elements are mapped to camera frame (CF)_L_:

1 $$ {}_{{\left( {\text{L}} \right)}} \varvec{P}_{{{\text{NP}},i}} = h\left( {\varvec{D}\left( {t_{0} } \right),{}_{{\left( {\text{LI}} \right)}} \varvec{p}_{{{\text{NP}},i}}  } \right) = \left( {{}_{\left( {\text{L}} \right)} x_{{\text{NP}},i} ,{}_{\left( {\text{L}} \right)} y_{{\text{NP}},i} ,{}_{\left( {\text{L}} \right)} z_{{\text{NP}},i}  } \right)^{\text{T}} \in {\mathbb{R}}^{3} , $$

where *h* describes the direct transformation ${\mathbb{R}}^{2} \to {\mathbb{R}}^{3}$ based on corresponding depth map $\varvec{D}\left( {t_{0} } \right)$ provided by scene reconstruction. Finally, concatenation of elements yields nominal path

2 $$ {}_{\left( {\text{L}} \right)} \varvec{t}_{\text{NP}} = \left( {{}_{\left( {\text{L}} \right)} \varvec{P}_{{\text{NP}},  1} ,{}_{\left( {\text{L}} \right)} \varvec{P}_{{\text{NP}},  i} ,\ldots,{}_{\left( {\text{L}} \right)} \varvec{P}_{{\text{NP}},M} } \right)^{\text{T}} \in {\mathbb{R}}^{3 \times M} . $$

Successful completion of the detection workflow is indicated to the operator. Data is stored persistently for post-experimental analysis. In case of initialisation errors, automatic re-initialisation and data validation are executed.

### Detection of Laser Spot Positions

After completion of nominal path detection, subjects activated teleoperation and completed the delineation task. The experimental assessment of the resulting tracing error demands for online detection and computation of the actual laser spot position on the tissue surface based on the workflow proposed in Fig. 3b.

The spot detection uses stabilised left camera images $\varvec{I}_{\text{LS}} \left( t \right)$ and corresponding depth map $\varvec{D}\left( t \right)$. Data sources are synchronised at time step *t* by approximate-time policies of the ROS framework. The experimental protocol ensures activation of the visible laser by the operator. Consequently, the centroid of the visible laser spot ${}_{{\left( {\text{LI}} \right)}} \varvec{p}_{\text{LC}}$ must be detected in image $\varvec{I}_{\text{LS}} \left( t \right)$. Robust detection of laser spots has been addressed in related work for example with template matching, modified Hough detectors, colour-based segmentation, Fourier and Zernike transforms, or neuronal networks.^9^,^32^

Based on related work, colour thresholding is considered for spot segmentation in image $\varvec{I}_{\text{LS}} \left( t \right)$ as part of the workflow shown in Fig. 3b. Frame-wise spot detection is further complemented by morphological operators for artefact removal and minimum area filter that yield binary map $\varvec{I}_{\text{BC}} \left( t \right)$. The latter is analysed with connected-component labelling to eliminate satellite areas. Valid regions must comply with following constraints: area size (>10 px) and circular shape conformity (>75%). Image moments enable estimation of laser centroid ${}_{{\left( {\text{LI}} \right)}} \varvec{p}_{\text{LC}} \left( t \right) = \left( {{}_{{\left( {\text{LI}} \right)}} u_{\text{LC}} \left( t \right),{}_{{\left( {\text{LI}} \right)}} v_{\text{LC}} \left( t \right)} \right)^{\text{T}} \in {\mathbb{N}}^{2}$. Temporal consistency is ensured by discarding frame-to-frame centre shifts of more than 5 px.

As depicted in Fig. 3b, segmentation and centroid estimation are executed in two consecutive steps to reject intermediate results if validation criteria are violated. Spot position ${}_{{\left( {\text{LI}} \right)}} \varvec{p}_{\text{LC}} \left( t \right)$ is mapped to frame (CF)_L_ by

3 $$ {}_{\left( {\text{L}} \right)} \varvec{P}_{\text{LC}} \left( t \right) = h\left( {\varvec{D}\left( t \right),{}_{{\left( {\text{LI}} \right)}} \varvec{p}_{\text{LC}} \left( t \right) } \right) = \left( {{}_{\left( {\text{L}} \right)} x_{\text{LC}} \left( t \right),{}_{\left( {\text{L}} \right)} y_{\text{LC}} \left( t \right),{}_{\left( {\text{L}} \right)} z_{\text{LC}} \left( t \right) } \right)^{\text{T}} \in {\mathbb{R}}^{3} , $$

where *h* defines the direct transformation ${\mathbb{R}}^{2} \to {\mathbb{R}}^{3}$ with depth map $\varvec{D}\left( t \right)$ provided by scene reconstruction. Lastly, laser centroids $\varvec{P}_{\text{LC}} \left( t \right)$ were processed at discrete measurement time steps $t_{k}$ and stacked to delineation trajectory

4 $$ \varvec{t}_{\text{LC}} = \left( {\begin{array}{*{20}c} {\varvec{P}_{\text{LC}} \left( {t_{1} } \right),\varvec{P}_{\text{LC}} \left( {t_{k} } \right), \ldots ,\varvec{P}_{\text{LC}} \left( {t_{N} } \right)} \\ {t_{1} ,t_{k} , \ldots ,t_{N} } \\ \end{array} } \right) \in {\mathbb{R}}^{4 \times N} $$

until the user completed the task and triggered abortion after *N* recorded samples. Trajectory $\varvec{t}_{\text{LC}}$ summarises temporal motion profiles of the teleoperated laser spot in frame (CF)_L_. Figure 3b further illustrates data validity assessment of the measurement workflow and decision policies if algorithmic constraints, e.g. due to erroneous spot detection or depth map, are violated.

### Evaluation Methodology

The evaluation of the study considers two metrics denoted as task completion time TCT and path tracing error PTE. Metric TCT determines the duration from laser activation and release of the master to deactivation of both features after task completion. Hence, this metric is defined as: ${\text{TCT}} = t_{N} - t_{1}$, where $t_{\text{N}}$ and $t_{ 1}$ describe the final and initial timestamp of corresponding laser centroids.

**Figure 4:**
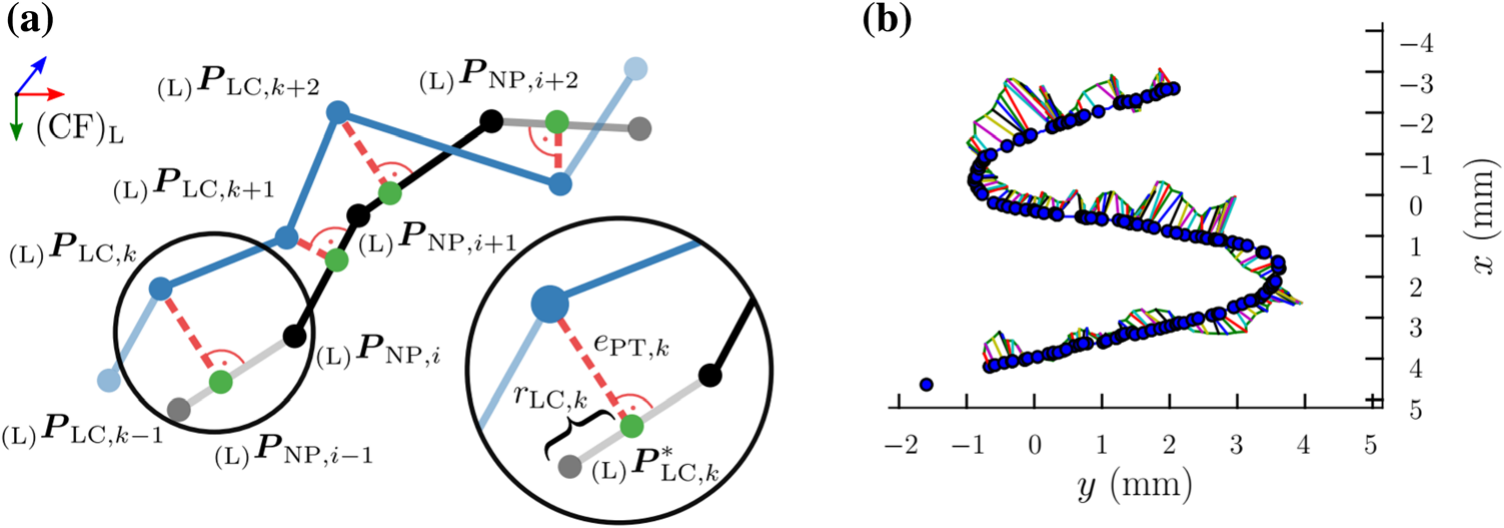
Evaluation workflow of the path tracing study: (a) Discrete representation and geometric annotation for PTE metric determination and (b) example of processed trajectory in x-y plane of (CF)_L_. Coloured features in (a) indicate the nominal path (black), measured laser spot positions (blue), orthogonal path errors (red), and projected positions (green). Circle marker in (b) denote a sparse set, i.e. only one-fifth of total data is depicted for visualisation purposes.

By contrast, computation of PTE requires multiple steps. Measurements of nominal path $\varvec{t}_{\text{NP}}$ and laser spot trajectory $\varvec{t}_{\text{LC}}$ are depicted in Fig. 4a. Metric PTE describes the geometric deviation between nominal path and recorded tracing trajectory. Common metrics for path similarity analysis are Hausdorff or Fréchet distances.^33^ In robotic laser surgery, orthogonal distances were established as gold standard.^19^ This approach is generalised from planar to 3D measurements.

Since both trajectories are given in common frame (CF)_L_, orthogonal projected positions of laser spot $\varvec{P}_{{{\text{LC}},k }}$ on segments constituting nominal path $\varvec{t}_{\text{NP}}$ are computed as illustrated in Figs. 4a and 4b:

$$ {\varvec{P}_{{{\text{LC}},k^*}}} = \varvec{P}_{{{\text{NP}},i}} + \left( {\varvec{P}_{{{\text{NP}},i + 1}} - \varvec{P}_{{{\text{NP}},i}} } \right)\frac{{\left( {\varvec{P}_{{{\text{LC}},k}} - {\varvec{P}_{{{\text{NP}},i}} }} \right)\left( {\varvec{P}_{{{\text{NP}},i + 1}} - \varvec{P}_{{{\text{NP}},i}} } \right)}}{{\frac{{\varvec{P}_{{{\text{NP}},i + 1}} - \varvec{P}_{{{\text{NP}},i_2}} }}{{r_{{{\text{LC}},k}} }}}} $$

where *i* $= \left\{ {1, \ldots ,\,M} \right\}$ and $k = \left\{ {1, \ldots ,\,N} \right\}$, respectively. If associated scaling factor $r_{{{\text{LC}},k}}$ satisfies condition $0 \le r_{{{\text{LC}},k}} \le 1$, the spot measurement is projected to the corresponding path segment and is taken into consideration for further processing. On the contrary, factor $r_{{{\text{LC}},k}}$ and correlated path segments were excluded if conditions are violated. This yields orthogonal path error of the *k*-th spot measurement and corresponding segmental projection on the nominal path:

5 $$ e_{{{\text{PT}},k}} = \|\varvec{P}_{{{\text{LC}},k}} - \varvec{P}^{*}_{{{\text{LC}},{k}}}\|_{2}.$$

Path errors of each pattern were composed to error vector $\varvec{e}_{\text{PT}} = \left( {e_{{{\text{PT}},1}} ,\,e_{{{\text{PT}},k}} , \ldots ,\,e_{{{\text{PT}},N}} } \right)^{\text{T}}$. with $k = \left\{ {1, \ldots ,\,N} \right\}$. The RMSE of $\varvec{e}_{\text{PT}}$ provides metric

6 $$ {\text{PTE}} = \sqrt {\frac{1}{N}\mathop \sum \limits_{k = 1}^{N} \left( {e_{{{\text{PT}},k}} } \right)^{2} } . $$

Computed PTE and TCT of each task and subject are consolidated for the entire study. Based on these measurements, mean, standard error, and median of study metrics are determined per subject. The 3D delineation case study additionally regards the focal position error FPE that is based on the Euclidean distance of pre-registered laser focus and nominal path as described in prior work.^17^

### Statistical Analysis

Statistics is applied to metrics PTE and TCT in order to identify effects of visualisation or subject-individual parameters. Both metrics are dependent variables that are complemented by three independent variables. The latter describe factors *experience*, *condition*, and *measurement*. Between-subject factor *experience* accommodates a two-level description of subject experience, i.e. novice or expert. Stabilised and non-stabilised visualisation *condition* yields a two-level within-subject factor decoded by acronyms NS and AS. Repeated measurements per subject are accommodated by within-subject factor *measurement* with five levels pursuant to number of trials per *condition*.

Statistical tests target to reveal significant metric differences grouped by factors. Primarily, all participants are considered as joint group for within-subject analysis only. Secondly, a mixed design consolidates within-subject factors and between-subject factor *experience*. Parametric multi-way analysis of variances (ANOVA) or non-parametric equivalents determine interaction effects. Data preparation regards four assumptions: (1) presence of outliers, (2) normal distribution of residuals, (3) equal variances of dependent variables (between-subject factors), and (4) equal variance of differences between factor-related groups.^11^ Residual normality is analysed with normal Q–Q plots and Shapiro–Wilk tests. Homoscedasticity is examined by Levene’s test. Mauchly’s test assesses equality of differences for within-subject factors.

## Porcine Model

**Figure 5:**
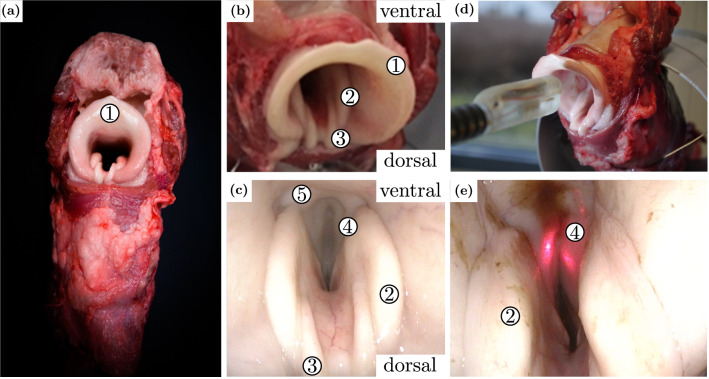
Anatomy of the porcine larynx: (a) dorsal view, (b) close-up of the anatomical differences, (c) view into the inner larynx. (d) Experimental setup and deployment of the endoscopic tip into the inner larynx. The specimen resides in a bespoke frame. (e) Visualization and tracing of the vocal folds. Anatomy annotation: 1. epiglottis, 2. paired arytenoid, 3. paired interarytenoid cartilages, 4. vocal fold, and 5. intralaryngeal fat pad.

The porcine larynx model is a validated specimen for surgical experiments due to the similarities to humans in anatomy, physiology, and functionality.^31^ Only minor anatomical variations are present in comparison to the human counterpart.^3^ The porcine epiglottis is defined by a wider shape and emanates in the cricoid cartilage front face. However, the main difference are the twin-paired arytenoid cartilages, which not only penetrate far more into the inner larynx but have a second pair of interarytenoid cartilages. Additionally, the porcine laryngeal lumen is further narrowed by an intralaryngeal fat pad (Figs. 5a–5c). For further examination of the system’s practicality, a simulated surgical setting was created using an *ex vivo* porcine larynx specimen. Objectives of this evaluation are specifically dedicated to landmark visualisation and manipulation of the endoscopic robot within the laryngeal framework. The specimen has been acquired from a local food processing facility shortly after the animal’s expiration and has been stored at 6°C for 12 h prior to experimental use. This *ex vivo* model respects ethical principles in animal research. To establish a resemblance to a patient lying on its back, as it would be in an actual surgery, the specimens with all their adjoining parts (tongue, epiglottis, outer/inner larynx and trachea) were positioned in a bespoke frame. The robotic system remained on its surgical cart as used in the previous experiment. This enables the system to approach the laryngeal specimen from the top. Each subject (*n* = 3) controlled the system with the haptic master. Study objectives were deployment of the endoscopic tip into the inner larynx (Figs. 5a and 5b), visualisation of relevant laryngeal landmarks in the endoscopic images, their robotic delineation using the pilot laser (Fig. 5c), and finally retraction of the system. Acquired video data was assessed post-experimentally by an expert not involved in the study.

## Results

### Delineation User Study

The user study comprised 20 subjects from two experience levels and multiple trials per subject and visualisation condition (*N *= 171). More precisely, 7 subjects (3 females, 4 males) from the Department of Otorhinolaryngology, Ulm University Medical Centre, Germany completed trials (*N *= 42) with an expert background in endoscopy as physicians and an age of 30.1 ± 4.9 years on average. On the other hand, 13 subjects (2 females, 11 males) from the Institute of Mechatronic Systems, Leibniz Universität Hannover, Germany equivalently completed trials (*N *= 129) without prior experience in endoscopy (novices) and an age of 28.1 ± 3.1 years on average. Measurements from both groups (*N *= 29) have been excluded from post-experimental analysis due to incomplete data acquisition. Visualisation of endoscopic images on the head-mounted display was preferred by 3 subjects, whereas 17 subjects selected the 3D monitor.

### Study Metrics

**Figure 6:**
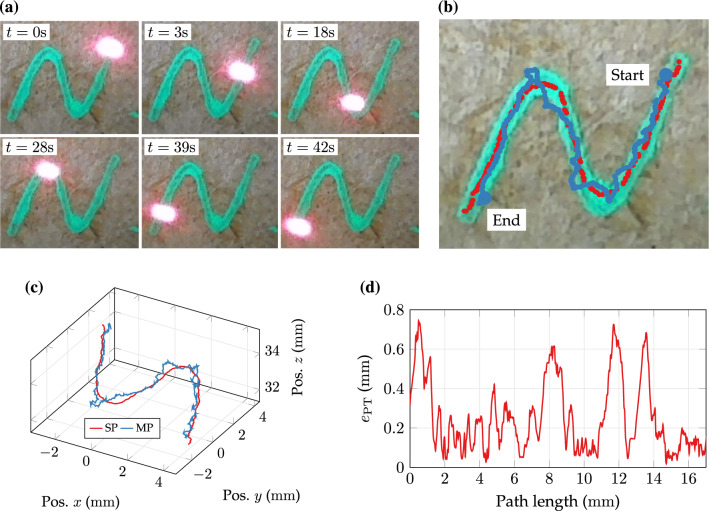
Example of evaluation workflow and study results for subject P18 and trial M10. (a) Temporal sequence of teleoperated tracing motion on S-shaped nominal pattern. (b) Post-experimental representation of automatically segmented nominal centreline (red) and measured laser spot positions (blue). (c) Depth map-based mapping of data in (b) to 3D space with reference to (CF)_L_. (d) Corresponding path tracing errors over length of nominal path.

An example of post-processing results according to the workflow described in “Image-Based Measurements” section is shown in Fig. 6. Images were evaluated and nominal path as well as laser spot locations were segmented (see Figs. 6a and 6b). Afterwards, image features are mapped to 3D space and path tracing errors $e_{\text{PT}}$ are computed for the entire path length as depicted in Figs. 6c and 6d.

The subject initially displaces the laser spot linearly from the start position towards the path centre line and $e_{\text{PT}}$<0.4 mm. While approaching a turn, tracing errors increase beyond 0.6 mm until the operator inputs corrective spot motions.

**Figure 7:**
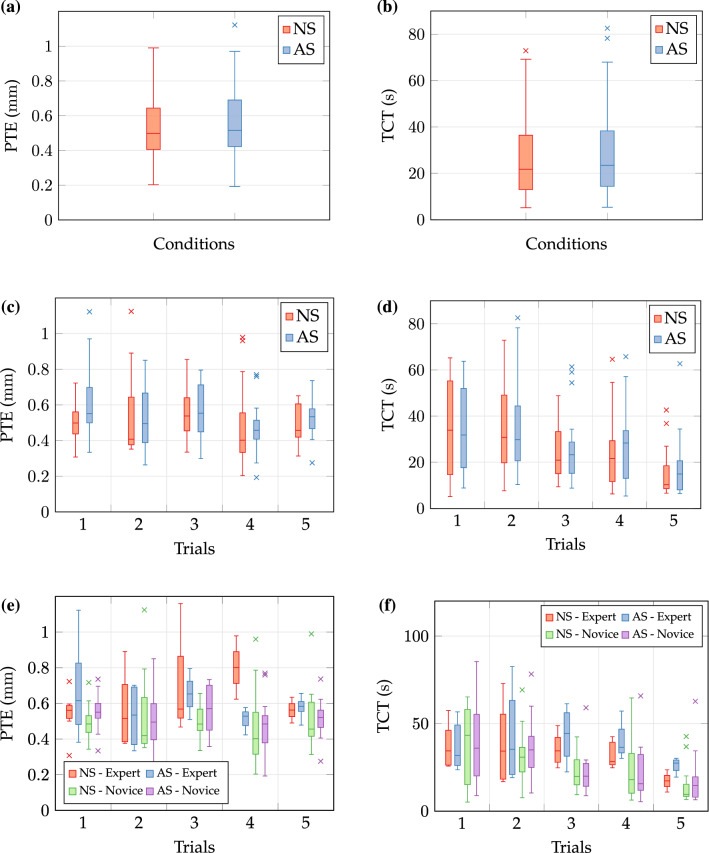
Box plots of metrics path tracing error (PTE) and task completion time (TCT) for non-stabilised (NS) and active scene stabilisation (AS): (a), (b) Condition-related results. (c), (d) Trial-resolved results. (e), (f) Condition-resolved split to subject groups. Horizontal dashes in the box depict the median and cross symbols indicate outliers. Boxes define 0.25 and 0.75-quantiles of input data. Upper and lower whisker span all data within 1.5 interquartile range of the nearer quartile.

Errors $e_{\text{PT}}$ and task completions times per subject are taken into consideration for computation of PTE and TCT. Metrics are consolidated to box plots (see Fig. 7) and summarised in Tables 2 and 3. Both task metrics show minor variations for visualisation conditions NS and AS. By contrast, an effect of repeated trials on completion time reduction is observable. Lastly, tracing errors and completion times are slightly increased for experts compared to novices. Statistical analysis was applied to identify effects on task metrics at significance level $\alpha$= 0.05. Primarily, a two-way repeated measures ANOVA with two-within factors was conducted. This considers within-subject factors *condition* and *measurement* only and disregards between-subject factor *experience*. PTE data was split into 10 subsets according to factorial combinations. After logarithmic transformation (right skewed distribution) Shapiro–Wilk tests validated subsets’ normality (*p *>* 0.*05). Mauchly’s test for factors *condition* and *measurement* revealed subset homogeneity: *measurement* (*W *= 0.17*, p *= 0.94), the interaction of *condition *× *measurement* (*W *= 0.27*, p *= 0.28), and directly for two level *condition*. There was no statistically significant two-way interaction of *condition* and *measurement* (*F*_4,40_ = 0.32*, p *= 0.86) nor main effects of *measurement* (*F*_4,40_ = 2.47*, p *= 0.06) and *condition* (*F*_4,40_ = 2.06*, p *= 0.18). There are no differences of PTE related to factors *measurement* and *condition*.

**Table 2:** Path tracing error metrics in mm per subject group and condition.

	NS			AS		
	Mean ± SD	Mdn	Max	Mean ± SD	Mdn	Max
	**0.55 **±**0.12**	0.50	0.90	0.56 ± 0.13	0.62	1.12
Expert	**0.62 **±**0.15**	0.56	0.90	0.66 ±0.10	0.67	0.87
Novice	0.53 ±0.08	0.53	0.75	**0.51 **±**0.14**	0.58	1.12

No image stabilisation (NS) and active image stabilisation (AS). Numbers marked in bold indicate the best performance per group

**Table 3:** Task completion time in s per subject group and condition.

	NS			AS		
	Mean ± SD	Mdn	Max	Mean ± SD	Mdn	Max
	**30.6 ± 14.2**	27.2	72.9	32.3 ± 16.7	28.4	82.6
Expert	**40.5 ± 17.6**	33.2	72.9	42.0 ± 21.7	41.0	82.6
Novice	**25.2 ± 7.7**	23.7	37.5	27.1 ± 10.0	22.5	50.8

No image stabilisation (NS) and active image stabilisation (AS). Numbers marked in bold indicate the best performance per group

A two-way repeated measures ANOVA was applied to metric TCT. Data was transformed logarithmically (right skewed distribution) and residual normality was verified with normal Q–Q plots and Shapiro–Wilk tests (*p *>* 0.*05). Mauchly’s test indicated sphericity for interaction of *condition *× *measurement* (*W *= 0.15*, p *= 0.08) and *measurement* (*W *= 0.23*, p *= 0.21). There was no statistically significant two-way interaction between *condition* and *measurement* (*F*_4,40_ = 0.21*, p *= 0.93) nor a main effect of *condition* on TCT data (*F*_1,10_ = 2.84*, p *= 0.12). On the contrary, a main effect of *measurement* indicated a statistically significant difference of TCT related to trials per subject for non-stabilised visualisation from the first trial to the last trial of 18.7 s and for stabilised visualisation of 20 s (*F*_4,40_ = 3.86*, p *= 0.009). Statistical results confirm preceding observations. There is no main effect of *condition* on TCT data, but the latter decreases significantly with increasing number of trials. Finally, between-subject factor *experience* was incorporated to previous within-subject designs yielding a three-way mixed ANOVA. Metric PTE of split subject groups was assessed and split to ten data subsets corresponding to factorial combinations. Data residuals satisfied normality after logarithmic transformation as assessed by normal Q–Q plots and Shapiro–Wilk tests (*p *>* 0.*05). Due to the mixed design, testing subset equality of variances for between-subject factors is required. Levene’s test demonstrated variance homogeneity (*F *= 0.06*, p *= 0.82) and Mauchly’s test confirmed sphericity for within-subject factors: *measurement* (*W *= 0.24*, p *= 0.15), interaction of *condition *× *measurement* (*W *= 0.34*, p *= 0.34), and inherently for *condition* with only two levels. There is no significant three-way interaction between within-subject factors *condition*, *measurement*, and between-subject factor *experience* (*F*_4,44_ = 0.71*, p *= 0.59). Likewise, two-way interactions of *experience *× *condition* (*F*_1,11_ = 1.55*, p *= 0.24), *experience *× *measurement* (*F*_4,44_ = 0.99*, p *= 0.42), and *condition *× *measurement* (*F*_4,44_ = 0.08*, p *= 0.99) were not statistically significant. Factors *measurement* (*F*_1,11_ = 2.27*, p *= 0.08) and *condition* (*F*_1,11_ = 1.13*, p *= 0.31) did not show main effects. On the contrary, factor *experience* shows a statistically significant main effect (*F*_1,11_ = 11.95*, p *= 0.005). This finding supports the observation of increased expert PTE compared to novices. Lastly, split subject groups TCT were analysed after logarithmic data transformation. Normality of residuals was proven by inspection of Q–Q plots and Shapiro–Wilk tests (*p *>* 0.*05). There was homogeneity of variances as assessed by Levene’s test (*F *= 1.03*, p *= 0.32) and Mauchly’s test confirmed sphericity for all within-subject factors: *measurement* (*W *= 0.43*,p *= 0.52), the interaction of *condition *× *measurement* (*W *= 0.21*, p *= 0.12), and directly for *condition* with two levels. There was no statistically significant three-way interaction (*F*_4,44_ = 1.34*, p *= 0.18), no two-way interaction of *background* × *condition* (*F*_4,44_ = 0.34*, p *= 0.37), nor main effects of *condition* (*F*_1,11_ = 2.34*, p *= 0.77) or *measurement* (*F*_1,11_ = 1.21*, p *= 0.53). However, there was a two-way interaction of *experience *× *measurement* (*F*_4,44_ = 6.21*, p *= 0.02). These results are consistent with observations of decreasing TCT throughout the trials.

### User Experience

**Figure 8:**
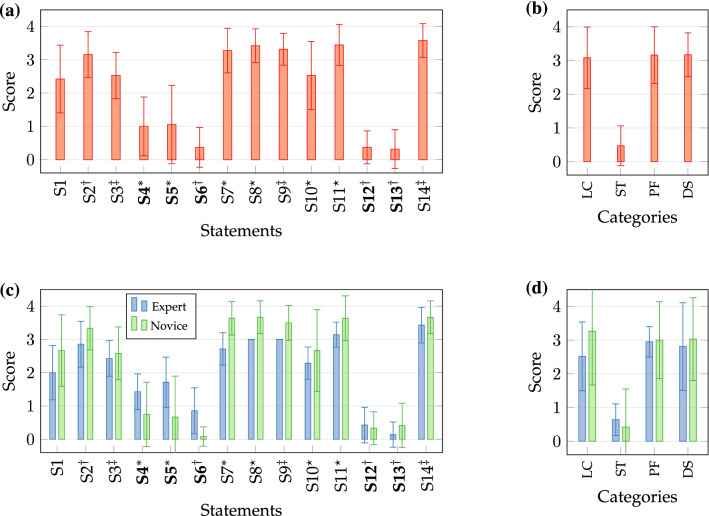
Scores of post-experimental user survey for path tracing study: (a) Results of individual statements and (b) grouped to categories. (c) Results of between-subject factor split to subject groups for individual statements and (d) grouped to categories. Category labels indicate learning curve (LC), stress (ST), system performance (PF), and system design (DS). Labels marked in bold indicate negative statements. Statements are linked by † to ST, ‡ to PF, * to LC, and ⋆ to DS.

The post-experimental survey is summarised in Fig. 8. Statements are consolidated to categories *learning curve* (S4, S5, S7), *stress* (S2, S6, S12, S13), *performance* (S3, S9, S14), and *design* (S8, S10, S11). Scores are provided in Figs. 8a and 8b related to statements and grouped to categories. The subjective assessment confirms results of quantitative metrics. Scores (mean ± SD) of category *learning curve* (3.08 ± 0.91) proof a fast familiarisation with the robotic system and successful task completion already after the first trial. Subjects were above average satisfied with their *performance* regarding accuracy and duration (3.16 ± 0.84) and acknowledged the integrated device *design* (3.17 ± 0.65). Use of the hardware has further exerted low *stress* (0.47 ± 0.59). Remarkably, scores of statement S1 do not indicate a preferred visualisation mode (2.42 ± 1.02) as both were rated equally. Results of split subject groups are presented in Figs. 8c and 8d. Slightly lower scores for experts compared to novices are observable. Inferential statistics were applied to data grouped by categories. Nonconformity to residual normality was indicated by Shapiro–Wilk tests (*p *<* 0.*05) and nonparametric Mann–Whitney-U test was considered. There were no statistically significant differences between mean populations of experts and novices for categories *performance* (*W *= 279*, p *= 0.07) and *stress* (*W *= 527*, p *= 0.08) nor related to statement S1 (visualisation condition) (*W *= 24*, p *= 0.1). However, significant differences are determined for *design* (*W *= 198*, p *= 0.002) and *learning curve* (*W *= 133*, p *<* 0.*001). This supports the observation of lower expert scores for device familiarisation.

### 3D Path Delineation Case Study

**Figure 9:**
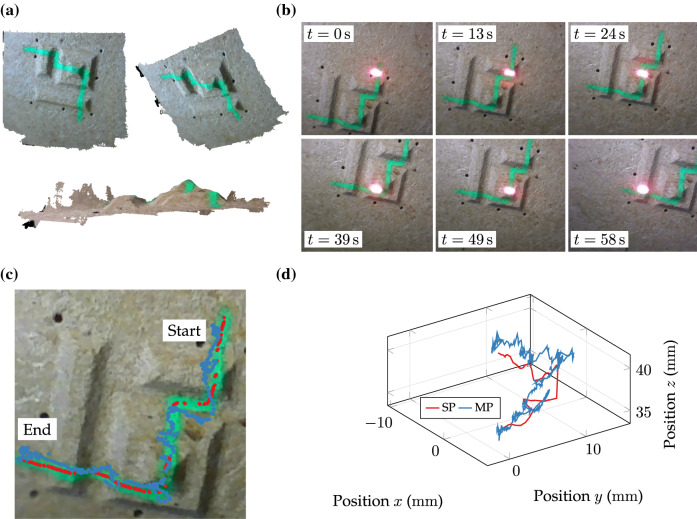
Example of 3D path delineation: (a) Specimen reconstruction with non-planar surface relief and coated path. (b) Temporal sequence of motion-compensated left camera view. (c) Post-experimental results of segmented nominal path (red) and projected spot measurements (blue). (d) Post-experimental spatial results of segmented nominal path (SP) and measured spot path (MP).

**Figure 10:**
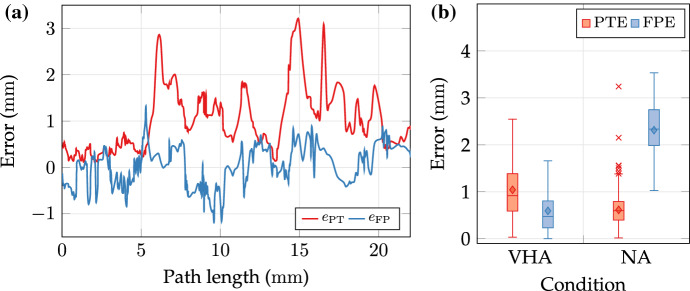
Results of 3D delineation case study: (a) Local path errors e_PT_ and focal errors e_FP_ along the nominal path with assistance. (b) Box plots of path tracing error (PTE) and focal position error (FPE) metrics for trials with visuo-haptic assistance (VHA) and no assistance (NA). Dashes in the box define the median, diamonds mark the mean, and cross markers indicate outliers. Boxes define 0.25 and 0.75-quantiles of input data. Upper and lower whisker span all data within 1.5 interquartile range of the nearer quartile.

Three subjects (research assistants, 29.3 ± 1.2 years on average) with advanced robotic training (> 5 h) completed three 3D delineation trials for each assistive condition (VHA/NA) on the pyramidal relief (*N* = 18). An example of a temporal sequence with activated assistance is shown in Fig. 9b. The laser spot is guided along the nominal path across the relief. Projections of nominal and measured paths are depicted in Fig. 9c. The corresponding spatial representation of segmentation and measurements is provided in Fig. 9d. The latter reveals spatial path differences composed of PTE and FPE contributions. A quantitative example of contributing errors along the path is shown in Fig. 10a. The FPE varies between ± 1 mm over the path length of approximately 22 mm. Results of preliminary trials on 3D beam manipulation are consolidated to Fig. 10b.

The PTE (mean ± SD) of condition VHA (1.04 ± 0.61) mm has been increased compared to condition NA (0.66 ± 0.29) mm. On the contrary, the FPE of condition NA (2.39 ± 0.51) mm is increased by a factor of four in comparison to VHA (0.59 ±0.51) mm.

### Porcine Model Deployment

Lastly, device deployment to the *ex vivo* porcine model was assessed. The porcine larynx was repeatedly explored with teleoperation by each expert subject (*n* = 3). In each trial, the deployment of the system into the porcine larynx was feasible. All relevant anatomical structures such as the laryngeal epiglottic surface, lateral, dorsal and ventral faces of the supraglottic inlet and especially the glottic region itself, could be visualised by the endoscopic imaging. The pilot beam was continuously perceivable and easily maneuverable by all subjects alongside the vestibular and vocal folds thus imitating a surgical manipulation as it would be conducted during endolaryngeal microsurgery (see Fig. 5e). A manipulation example is provided by sequence C of the supplemental video.

## Discussion

Two commercial robotic systems, DaVinci® and Flex®, are currently approved for TORS. Both systems have proven feasibility in endolaryngeal surgery. Alternatively, research devices were assessed in preclinical settings. However, TORS has not been established in clinical routines due to financial burdens, prolonged setup times, limited dexterity, and lack of patient benefits.^23^ To target these shortcomings, we present the performance evaluation of a novel endoscopic robot for non-contact laser surgery and its preliminary deployment to a porcine *ex vivo* model. Besides its integrated laser beam and unlike devices in literature, the system features full 3 DoF master–slave teleoperation with motion-compensated endoscopic views to accommodate tip motions. To the present day, the use of laser instrumentation with TORS systems remains challenging as dexterous manipulation with high accuracy and continuous adjustment of optical components is mandatory to minimise tissue trauma. Those problems have been addressed in this study and our previous work on user assistance.^17^ Conversely, the advantages of dexterous endoscopic beam manipulation are presented in a user-centred assessment of the robotic performance with delineation tasks. Tracing RMSE of below 0.75 mm were achieved by most subjects for both visualisation modes. Remarkably, error outliers are limited. These findings correlate with accuracy of extracorporeal scanners.^10^ The results further demonstrate that tracing errors were not significantly biased by the number of trials, whereas TCT diminished gradually to one-third. This supports effective device familiarisation with steep learning curves. Low tracing errors were already obtained after induction as substantiated by survey results on device design. Errors are on average slightly higher for motion-compensated than raw endoscopic visualisation. This also applies to TCT for both subject groups. This is mainly due to latencies (10-30 ms) caused by the algorithmic processing and user visualisation framework. Accordingly, subjects reduced manipulation velocities after familiarisation with the robotic device and view compensation for accurate tracing of delicate sections in the given task. The deferred visual feedback on actual laser positions and corresponding teleoperation input results in minor deviations. The survey supports prospective consideration of motion-compensated endoscopic views, despite novices facilitate slightly lower errors than experts. We assume that prior familiarisation to endoscopic equipment demands for adaptation to novel input strategies. Surgical trainees and novices are probably more adapted to electronic devices and may have less difficulties in integrating robotics into a surgical workflow, e.g. surgical skill improvement from regular videogaming.^20^

The case study on full 3D manipulation has demonstrated that trained operators successfully completed delineation tasks on non-planar surfaces. It was shown that consideration of assistive features whilst completing the delineation task can significantly improve the adjustment of the focal distance for non-contact application. This enables clinicians to complete complex interventions without iterative adjustments of focal settings during the procedure. However, this currently goes along with increased path tracing errors as lateral operator input may be affected by cross effects from assistive methodologies. This assumption is supported by observation of reduced PTE for absence of assistance. Those study results are further confirmed by device deployment to the *ex vivo* porcine larynx. All anatomical landmarks were exposed and outlined by subjects. The successful exposure motivates to include and augment preoperative imaging to the endoscopic live view in order to improve the localisation of lesions. Nonetheless, the porcine model was reduced to the laryngeal framework to evaluate the intralaryngeal manipulation. Insertion to the laryngeal space was not in the scope of this study.

Current limitations of the proposed platform are: (1) End-effector velocity limitation of 3.5 mm s^−1^ and manipulation in three linear DoF only to ensure structural integrity, robust visual processing, and controller stability. This prevents TCT reduction and prospectively impacts laser settings for optimal pulse overlaps. (2) Online vision processing and sensor acquisition (25 Hz) introduce latencies that must be optimised for system dynamics and stability. Minor stick–slip phenomena originating from actuation tube guidance can cause minor tip vibrations and impacts subjects’ perception. Mitigation is expected from additional tubular coatings. (3) Reconstruction and tracking are both subject to systematic RMSE of approx. 0.1 mm.

### Conclusion

The availability of robotic platforms for dexterous luminal non-contact laser delivery under consideration of clinical challenges, such as needed for endolaryngeal surgery, is limited to the present day. This work describes implementation and performance evaluation of an alternative robotic platform for ablation in confined anatomy based on an extensible continuum robot. A comprehensive user study addressing novices and experts has verified our research hypothesis of accurate laser displacement with errors of less than 1 mm. Further on, subjects with advanced robotic training have proven feasibility of assisted laser focus manipulation on non-planar specimens. Device deployment to a porcine larynx has demonstrated manipulation in confined anatomy. Results support study objectives and further pave the way to *in vivo* endoscopic laser delivery.

Future work focuses on hardware miniaturisation, integration and impact of angular DoF with advanced haptic masters, design optimisation for friction elimination, and integration of high-power lasers. Imaging sensors with acquisition rates beyond 50 Hz, minimal latencies, and high definition may further optimise reconstruction and tracking. Further system evaluation aims at 3D manipulation on *ex vivo* tissue surfaces to complement results of the case study. Experimental studies with high-power laser ablation in *ex vivo* porcine models with residual oropharynx or cadaver models are targeted to assess feasibility of the complete deployment workflow of the robotic device, i.e. insertion to the laryngeal space. Lastly, further clinical applications with surgical access through natural orifices are explored that may target transanal or gastrointestinal laser interventions to treat haemorrhage and mucosal lesions.

## Electronic supplementary material

Below is the link to the electronic supplementary material.

Electronic supplementary material 1 (MP4 5355 kb)

## References

[1] Acemoglu A, Deshpande N, Mattos LS (2018). Towards a magnetically-actuated laser scanner for endoscopic microsurgeries. J. Med. Robot. Res..

[2] Adelman MR, Tsai LJ, Tangchitnob EP, Kahn BS (2013). Laser technology and applications in gynaecology. J. Obstet. Gynaecol..

[3] Alipour F, Finnegan EM, Jaiswal S (2013). Phonatory characteristics of the excised human larynx in comparison to other species. J. Voice.

[4] Alon EE, Kasperbauer JL, Olsen KD, Moore EJ (2012). Feasibility of transoral robotic-assisted supraglottic laryngectomy. Head Neck.

[5] Bajo A, Simaan N (2012). Kinematics-based detection and localization of contacts along multisegment continuum robots. IEEE Trans. Robot..

[6] Blanco RG, Ha PK, Califano JA, Saunders JM (2011). Transoral robotic surgery of the vocal cord. J. Laparoendosc. Adv. Surg. Tech..

[7] Blumenkranz MS (2014). The evolution of laser therapy in ophthalmology. Am. J. Ophthalmol..

[8] Chawla S, Carney AS (2009). Organ preservation surgery for laryngeal cancer. Head Neck Oncol..

[9] Cui JW, Tan JB, Ao L, Kang WJ (2005). Optimized algorithm of laser spot center location in strong noise. J. Phys..

[10] Dagnino G, Mattos LS, Caldwell DG (2015). A vision-based system for fast and accurate laser scanning in robot-assisted phonomicrosurgery. Int. J. Comput. Assist. Radiol. Surg..

[11] Feys J (2016). Nonparametric tests for the interaction in two-way factorial designs using R. R J..

[12] Friedrich DT, Scheithauer MO, Greve J, Hoffmann TK, Schuler PJ (2017). Recent advances in robot-assisted head and neck surgery. Int. J. Med. Robot..

[13] Gilling PJ, Fraundorfer MR (1998). Holmium laser prostatectomy: a technique in evolution. Curr. Opin. Urol..

[14] Hart SG, Staveland LE, Hancock PA, Meshkati N (1988). Development of NASA-TLX (task load index): results of empirical and theoretical research. Adv. Psychol..

[15] Kundrat, D., A. Fuchs, A. Schoob, L. Kahrs, T. Ortmaier. Endoluminal non-contact soft tissue ablation using fiber-based Er:YAG laser delivery. In: Proceedings of SPIE BiOS 9702, 2016.

[16] Kundrat D, Schoob A, Kahrs LA, Ortmaier T, Verl A, Albu-Schaffer A, Brock O, Raatz A (2015). Flexible robot for laser phonomicrosurgery. Soft Robotics.

[17] Kundrat D, Schoob A, Piskon T, Graesslin R, Schuler PJ, Hoffmann TK, Kahrs LA, Ortmaier T (2019). Toward assistive technologies for focus adjustment in teleoperated robotic non-contact laser surgery. IEEE T. Med. Robot. Bionics.

[18] Lewis JR (1991). An after-scenario questionnaire for usability studies: psychometric evaluation over three trials. SIGCHI Bull..

[19] Mattos LS, Deshpande N, Barresi G, Guastini L, Peretti G (2014). A novel computerized surgeon-machine interface for robot-assisted laser phonomicrosurgery. Laryngoscope.

[20] Moglia A, Perrone V, Ferrari V, Morelli L, Boggi U, Ferrari M, Mosca F, Cuschieri A (2017). Influence of videogames and musical instruments on performances at a simulator for robotic surgery. Minim. Invasive Ther. Allied Technol..

[21] Patel, S., M. Rajadhyaksha, S. Kirov, Y. Li, R. Toledo-Crow. Endoscopic laser scalpel for head and neck cancer surgery. In: Proceedings of SPIE BiOS 8207, 2012.

[22] Renevier R, Tamadazte B, Rabenorosoa K, Tavernier L, Andreff N (2017). Endoscopic laser surgery: design, modeling, and control. IEEE ASME Trans. Mech..

[23] Rudmik L, An W, Livingstone D, Matthews W, Seikaly H, Scrimger R, Marshall D (2015). Making a case for high-volume robotic surgery centers: a cost-effectiveness analysis of transoral robotic surgery. J. Surg. Oncol..

[24] Schindler A, Pizzorni N, Mozzanica F, Fantini M, Ginocchio D, Bertolin A, Crosetti E, Succo G (2016). Functional outcomes after supracricoid laryngectomy: what do we not know and what do we need to know?. Eur. Arch. Otorhinolaryngol..

[25] Schoob A, Kundrat D, Kahrs LA, Ortmaier T (2016). Comparative study on surface reconstruction accuracy of stereo imaging devices for microsurgery. Int. J. Comput. Assist. Radiol. Surg..

[26] Schoob A, Kundrat D, Kahrs LA, Ortmaier T (2017). Stereo vision-based tracking of soft tissue motion with application to online ablation control in laser microsurgery. Med Image Anal.

[27] Schoob A, Laves MH, Kahrs LA, Ortmaier T (2016). Soft tissue motion tracking with application to tablet-based incision planning in laser surgery. Int. J. Comput. Assist. Radiol. Surg..

[28] Schuler PJ (2018). Robotische Chirurgie – operiert der Roboter ? Robotic surgery – who is the boss ?. Laryngorhinootologie.

[29] Schuler PJ, Duvvuri U, Friedrich DT, Rotter N, Scheithauer MO, Hoffmann TK (2015). First use of a computer-assisted operator-controlled flexible endoscope for transoral surgery. Laryngoscope.

[30] Strong MS, Jako GJ (1972). Laser surgery in the larynx early clinical experience with continuous CO_2_ laser. Ann. Otol. Rhinol. Laryngol..

[31] Swindle MM, Makin A, Herron AJ, Clubb FJ, Frazier KS (2012). Swine as models in biomedical research and toxicology testing. Vet. Pathol..

[32] Vazquez-Otero A, Khikhlukha D, Solano-Altamirano JM, Dormido R, Duro N (2016). Laser spot detection based on reaction diffusion. Sensors.

[33] Youngung S, McMains S (2004). Evaluation of drawing on 3D surfaces with haptics. IEEE Comput. Graph. Appl..

[34] Zhang TY, Suen CY (1984). A fast parallel algorithm for thinning digital patterns. Commun. ACM.

